# The effect of physical activity on anxiety symptoms among children and adolescents with mental health disorders: a research brief

**DOI:** 10.3389/fpsyt.2024.1254050

**Published:** 2024-05-13

**Authors:** Ella Aase Anker, Svanhild E. Bøe Sture, Sigurd William Hystad, Arne Kodal

**Affiliations:** ^1^ Department of Child and Adolescent Psychiatry, Division of Psychiatry, Haukeland University Hospital, Bergen, Norway; ^2^ Department of Psychosocial Science, Faculty of Psychology, University of Bergen, Bergen, Norway; ^3^ Regional Centre for Child and Youth Mental Health and Child Welfare, NORCE Norwegian Research Centre, Bergen, Norway

**Keywords:** anxiety, physical activity, mental health, child & adolescent psychiatry, social phobia

## Abstract

**Background:**

Anxiety is prevalent among children and adolescents (termed youths), and leads to reduced quality of life, disability, loss of education and reduced life-span. Physical activity has shown promising effects on symptoms of anxiety in adult populations, and an increasing amount of research has also demonstrated some effect in youth. However, physical activity is not widely used in youth mental health care, and research is very limited.

**Methods/design:**

This single arm, pre-post study explores the effect of a manualized physical activity-based 14-session intervention termed Confident, Active and Happy Youth. Participants are youth attending specialized mental health care (*N*=51, *M* age = 13.4, *SD* = 2.2). Changes in anxiety symptoms are examined using mixed models with residual maximum likelihood (REML). The potential effect of anxiety subtype differences, participant age, comorbidity, and time spent in out-patient care are explored.

**Results:**

Youths did not report any effect on anxiety symptoms after participation in CAHY, however, their parents report a significant reduction in youth’s anxiety symptoms in general (b = -0.11, 95%, CI: -0.21 to -0.01) and specifically for social phobia (b = -0.19, 95% CI: -0.35 to -0.03). Age and comorbidity showed no significant effect on anxiety symptoms post treatment. Prior treatment time in youth mental health care demonstrated inconclusive results.

**Conclusion:**

The study finds initial evidence of symptomatic change in a clinical population of youth’s receiving a physical activity-based intervention. Our research provides preliminary support for physical activity as a supplementary treatment method for mental health disorders among youths.

**Clinical Trial Registration:**

clinicaltrials.gov, NCT05049759

## Introduction

Anxiety and depression are highly prevalent disorders among children and adolescents, and among the top five causes of overall disease burden among youth in Europe (1). These disorders typically develop before the age of 14 (2) and are associated with a range of short- and long-term consequences, including reduced quality of life, increased psychiatric and somatic comorbidity, disability, loss of education and eventually work, suicide and reduced life-span (3, 4). Rates of anxiety and depression in children and adolescents have increased following the Covid-19 pandemic (5) due to factors such as social isolation, (6), time away from school and decreased physical activity (7, 8). Not surprisingly, the current situation among children and adolescents is regarded as a major health crisis (9). To make matters worse, even when provided best available treatment for anxiety and depression is provided, youth post-treatment remission rates remain just slightly above chance e.g., 50% (10). Thus, development of new evidence-based approaches and/or supplementary interventions to help both mitigate the consequences of anxiety and depression in children and adolescents, improve recovery rates and prevent relapse is imperative (11, 12).

A viable and promising treatment avenue that may address the current critical situation is the use and integration of physical activity in the treatment of youth mental health disorders. In adult populations, physical activity is demonstrated to be efficacious for improving symptoms of anxiety and depression (13), and is in some cases even more beneficial in treating anxiety and depression than medication and standard psychotherapy alone (14). In terms of different types of anxiety disorders, panic disorder is the most frequently studied and has thus far demonstrated the most promising results when treated with physical activity interventions in combination with cognitive behavior therapy (13), although with varying results (13–15). Merom et al. (15), found reduction of anxiety symptoms after an intervention based on home- walking programs (moderate intensity) in addition to group cognitive behavior therapy on symptoms of social phobia. However, the same effect in participants diagnosed with panic disorder or generalized anxiety disorder was considered more uncertain.

In child and adolescent populations, there is substantially less research on the effect of physical activity than for adult populations in general, and to our knowledge no studies have examined variations in treatment response for different subtypes of anxiety. Most children experience universal fears at various stages of development (16). Separation fears for instance, are a normal part of an infant’s development as the child learns object permanence. If these fears are excessive and impinge on function, the child may develop a separation anxiety. Adolescents on the other hand typically are more concerned with social adjustment, and therefore social phobia is more prevalent at this age (17). More generally, several calls have been made for more research on the effect of physical activity on anxiety symptoms in children and adolescents (18, 19). A recent meta-analysis examined the effect of physical activity interventions on anxiety symptoms in adolescents and young adults (age < 25 years) and found a moderate effect on state anxiety (20), whereas meta-analyses on the effect of physical activity on depression have demonstrated small to moderate effects (21–23). Further, a recent study investigated the impact of physical exercise on anxiety and depression in adolescent inpatients and found that exercise had a moderate effect on depressive symptoms, although no effect on anxiety symptoms (24). Importantly, while current findings lend some support for the use of physical activity towards anxiety and depressive symptoms, the evidence is still limited and the general quality of studies is low (20, 21). Furthermore, apart from the study by Phillipot and colleagues, studies primarily investigated community samples even though physical activity may be particularly effective for clinical populations (20, 25). Physical activity-based interventions are per today not an integrated part of child and adolescent mental health care, even though physical activity is indicated as a key modifiable variable towards good mental health (25). Thus, there is a clear need for more research on the use of physical activity in the treatment of youth anxiety and depression, particularly within clinical populations.

On this backdrop, Haukeland University hospital has developed a trans-diagnostic physical- activity based supplemental treatment approach, targeting youth with anxiety and depressive symptoms, named Confident, Active and Happy Youth (CAHY). This treatment approach has undergone feasibility testing with results demonstrating that the approach is feasible (26). However, results from the trial demonstrate that the treatment leads to negligent changes on anxiety symptoms at post-treatment with the exception of parent-reported youth anxiety symptom reduction at 10-months follow-up (26). This warrants further examination.

Thus, in line with *The British Medical Research Council Guidance* (27) and towards the goal of conducting a definitive trial of the treatment, it is of particular interest to further explore both the effects of the physical activity treatment on anxiety symptoms and to explore if subgroups of anxiety respond differently to treatment, and whether age, psychiatric comorbidity and prior treatment time in a mental health clinic influences outcomes. This examination may tease out details regarding the need to either adjust the treatment to accommodate anxiety subgroups, and/or adjust participant inclusion criteria. Thus, the aim of the present study is to explore anxiety symptom changes following participation in a physical activity-based intervention among children and adolescents in treatment for mental illness in specialist health services.

Our study aims to answer the following research questions:

1) Does participation in the CAHY treatment program lead to changes in anxiety symptoms in children and adolescents?
2) Does age, comorbidity, and prior treatment time in Child and Adolescent Mental Health Services influence treatment symptom-change at post-treatment?

Given our previous findings (26), and lack of prior research on this topic, we did not state any *a priori* hypothesis regarding possible outcome.

## Methods

Confident Active Happy Youth (CAHY) was evaluated using a single arm, pre-post study which took place from August 2020 to March 2022, including baseline and post-intervention assessment. The study is a secondary analysis based on a prior feasibility trial (26) yet extended in terms of time so as to include more participants than the main feasibility study, which included 19 participants. Given the exploratory nature of the study, resource and time constraints, a convenience sample was used. Nevertheless, this population was assessed large enough to address the study goals.

## Participants

Inclusion criteria for participants were:

∘ Age 8-17 years.
∘ Symptoms of anxiety and/or depression assessed by the referring therapist.
∘ Youth displaying reduced daily physical activity (less than 30 mins. per day and/or does not partake in physical leisure activities, and/or does not participate in physical education in school).

Exclusion criteria were:

∘ Physical activity was not advised for medical reasons.
∘ Severe learning disabilities and the youth was unable to understand the intervention instructions (e.g., severe learning disabilities).
∘ Severe psychiatric disorders such as eating disorders and psychosis.
∘ Severe challenging behavior or other needs making group participation challenging.

### Participants and procedure

Youth were recruited from Child and Adolescent Mental Health Services (CAMHS), Department of Child and Adolescent Psychiatry, Haukeland University Hospital, Norway. Participants were referred from one of the seven outpatient clinics in CAMHS catchment area. CAMHS is the youths’ primary place of mental health treatment, including problem identification (why they are referred to CAMHS), diagnostic clarification and treatment. Thus, all clinical diagnoses a youth may qualify for, are assessed, and assigned by their attending clinical therapist and the responsibility for overall mental health follow-up rests with the clinician. Information about the intervention, inclusion and exclusion criteria was prior to the study distributed to all therapists in the outpatient clinics, and referring clinicians were encouraged to contact the CAHY team in case of any uncertainty regarding participant eligibility. Based on the CAMHS clinician’s clinical assessment of eligibility, a formal referral was sent to the CAHY team, whom then with the Principal Investigator (PI) assessed study eligibility. If assessed eligible, youth and their primary caregivers were sent an invitation to attend a pre-treatment assessment interview.

Informed written consent was obtained from all parents and youth aged 16 or above in the pre-treatment assessment interview, whereas assent was obtained from youth who were 12 years old or more. All participants could withdraw their consent with no consequences for continued participation (28). In the pre-treatment assessment, demographic data were gathered and questionnaires were filled out including SCAS-C/P. Post-treatment, participants were invited to attend a post-treatment assessment interview. In this interview participants and parents filled out the SCAS anew.

The participants (*N* = 51) consisted of 29 girls and 22 boys. Mean age while in the treatment program was 13.4 (*SD* = 2.2), with an age-range between 9 to 17 years. Average prior treatment time in CAMHS was 21 months (*SD* = 16.8) varying between two months to almost eight years (95 months). Given that the this was a clinical sample, all participants suffered from one or more psychiatric disorders. Thus, 43% had only one psychiatric diagnosis whereas 48% had at least one comorbid mental disorder (*M* = 0.96, *SD* = 1.0). Approximately half of the sample (45%) was diagnosed with an anxiety disorder (F40.0-42.9 in ICD-10 diagnostic system), and most prevalent was social phobia which accounted for 22%. One in four patients was diagnosed with depression (F32.0-33.9 in ICD-10) and 45% percent was diagnosed with ADHD. Importantly, an anxiety or depressive disorder was not a prerequisite for eligibility (see inclusion criteria), however all participants qualified for anxiety symptoms (51%), depression symptoms (12%) or both anxiety and depressive symptoms (37%).

### The intervention

CAHY was developed as a supplemental manualized trans-diagnostic treatment to existing interventions at the Child and Adolescent Psychiatric Mental Health Services (CAMHS) at Haukeland University Hospital with the goal of improving treatment outcome. CAHY is based on established evidence, concerning the effects of physical activity on mental health in youth specifically anxiety and depression, self-determination theory and inhibitory learning theory, combining these elements in a new approach (28). The intervention targets interrelated core symptoms of anxiety and depression in the form of reduced and/or low levels of physical activity, lack of confidence in one’s ability to tackle and cope with situations that incite distress and discomfort and lowered mood (28). The intervention aims to alleviate these core disorder features supplementing ongoing treatment, which translates to helping youth become more confident, active and happy, in short CAHY. The intervention is offered in age adjusted groups of a maximum of eight: a child group aged 8-12 years and adolescents group aged 13-17 years. Treatment sessions take place twice a week, are 50 minutes long, with the exception of the last session which is a three-hour long hike. The duration of the intervention is seven weeks. Sessions (with exception to the last session, which is outdoors) follow the same structure, and include a mix of primarily aerobic (e.g., running, jumping, traversing an obstacle course), resistance (e.g., squats, push-ups) and some relaxation exercises (e.g., yoga exercises). The physical activities are designed to stimulate some distress, fear and/or discomfort, which is coupled with psychoeducation regarding the bidirectional interplay between thoughts, feelings, actions and particularly the role of avoidance and alternative coping strategies. Thus, the physical activities also serve as an *in-vivo* exposure arena for the children and adolescents to learn new coping strategies.

### Measures and materials

Background variables including age, comorbidity (defined as number of diagnosis), and prior treatment time in CAMHS were gathered at pre-treatment assessment. Participant’s mental health status (i.e., psychiatric diagnosis/diagnoses) and treatment time and treatment status was procured from the participant’s medical records in CAMHS.

*Anxiety* was measured using Spence Children’s Anxiety Scale (SCAS) (29). Both the child version and parent version were used in order to enable a comparison between self- and external assessment. SCAS consist of six subscales including separation anxiety, social phobia, obsessive-compulsive disorder, panic/agoraphobia, generalized anxiety and fears of physical injury. The participant is asked to rate on a 4-point Likert scale: ‘never’, ‘sometimes’, ‘often’, or ‘always’ (scored 0-3) to indicate how often each of the items happens to them, and with a possible maximum score of 114. SCAS child-version (SCAS-C) consists of 45 items and SCAS parent-version (SCAS-P) consists of 39 items, of which 38 items in both versions are scored. We computed mean scores rather than sum scores for the total scales and subscales to allow for some missing responses and hence include as many participants as possible in our analysis. A minimum of 70% of the items within a subscale had to be completed. Cronbach’s alphas (α) are presented in **Tables 1**, **2**.

**Table 1:** Child reported Spence Children’s Anxiety Scale (SCAS-C) estimated total and subscale scores (means and standard errors).

Variables	n	# observation	Pre		Post		*t*	*p*
			M (SE)	α	M (SE)	α		
SCAS total	51	87	1.15 (0.06)	.90	1.10 (0.06)	.93	-1.19	.241
Separation anxiety	51	88	0.93 (0.08)	.75	0.88 (0.08)	.74	-0.88	.387
Social phobia	51	85	1.60 (0.09)	.82	1.46 (0.10)	.78	-1.76	.087
Obsessive compulsive	51	84	1.04 (0.09)	.69	1.04 (0.09)	.85	0.07	.941
Panic/agoraphobia	50	85	0.92 (0.08)	.82	0.88 (0.09)	.88	-0.59	.556
Physical injury fears	51	87	1.02 (0.07)	.26	0.98 (0.07)	.39	-0.74	.465
Generalized anxiety	50	85	1.38 (0.09)	.83	1.32 (0.09)	.84	-0.76	.451

SCAS-C, Spence Children’s Anxiety Scale – Child Version.

**Table 2:** Parent reported Spence Children’s Anxiety Scale (SCAS-P) estimated total and subscale scores (means and standard errors).

Variables	n	# observation	Pre		Post		*t*	*p*
			M (SE)	α	M (SE)	α		
SCAS total	50	91	1.05 (0.06)	.90	0.95 (0.06)	.92	-2.21	.032
Separation anxiety	51	93	1.08 (0.09)	.75	0.99 (0.09)	.80	-1.26	.215
Social phobia	51	88	1.70 (0.09)	.75	1.51 (0.09)	.79	-2.37	.023
Obsessive compulsive	50	90	0.84 (0.07)	.73	0.71 (0.07)	.70	-1.90	.064
Panic/agoraphobia	50	91	0.76 (0.08)	.85	0.66 (0.08)	.85	-1.49	.144
Physical injury fears	51	94	0.98 (0.07)	.31	0.91 (0.08)	.48	-1.23	.225
Generalized anxiety	50	92	1.12 (0.08)	.69	1.05 (0.08)	.79	-1.15	.256

SCAS-P, Spence Children’s Anxiety Scale – Parent Version.

### Statistical analyses

Mixed-effects models with residual maximum likelihood (REML) estimation were used to compare SCAS at pre- and post-measurement. We further used a small-sample adjustment for the hypothesis tests known as a Kenward-Roger approximation (30). Since mixed-effects models are fit with maximum likelihood, they do not require balanced data but instead use all available data on each participant. Under the assumption of missing at random (MAR), mixed models are expected to provide unbiased estimates (31, 32).

Separate mixed-effect models were run for total SCAS and the six anxiety domains as response variables. Differences between pre- and post-treatment scores were computed by including time as a fixed effect in the models. The effects of age, comorbidity, and time spent in out-patient care prior to participating in CAHY were explored by including interactions between time and the three variables in the models.

The mixed-effects models were conducted using STATA version 17.0 (33).

## Results

The results from the mixed-effects models are summarized in **Table 1** (child ratings) and 2 (parent ratings). None of the analyses involving child ratings revealed any statistically significant differences between pre- and post-treatment measurements of SCAS (see **Table 2**). There were main effects of age on total SCAS (*b* = 0.06, *p* = .03), Social phobia (*b* = 0.12, *p* <.01), obsessive compulsive disorder (*b* = 0.08, *p* = .04), Panic/agoraphobia (*b* = 0.11, *p* <.01), and Generalized anxiety (*b* = 0.10, *p* = .01). The positive regression coefficients imply that older participants reported higher levels of total SCAS and the four anxiety domains across the two measurement points.

**Figure 1:**
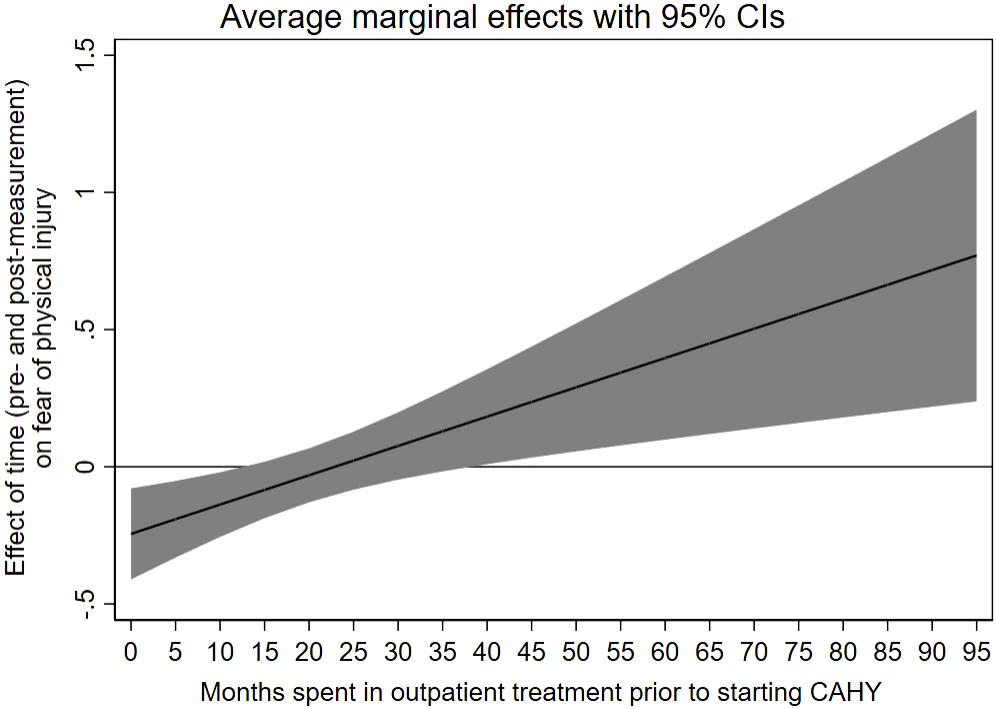
Association between prior treatment time in CAMHS and the subscale “fear of physical injury” on SCAS (child ratings).

The analyses that included interaction terms between measurement point and age, comorbidity, and time in treatment prior to starting CAHY revealed a statistically significant interaction between measurement point and time in treatment for fear of physical injury, *b* = 0.01, *p* <.01. This interaction is illustrated in **Figure 1** and shows that participants who spent relatively few months in outpatient care prior to CAHY are estimated to reduce their scores from pre- to post-measurement. As the number of months in outpatient care increases, the difference between pre- and post-measurement scores moves towards zero and into positive numbers (i.e., increases in scores from pre- to post measurement). Importantly, the parameter estimates get less precise and the confidence intervals broadens as the duration in outpatient care increases.

For parent ratings, there was a statistically significant reduction in total SCAS from pre- to post-measurement, *b* = -0.11, 95% confidence interval: -0.21 to -0.01. Analyses of subscale response showed that only social phobia decreased significantly, *b* = -0.19, 95% confidence interval: -0.35 to -0.03.

**Figure 2:**
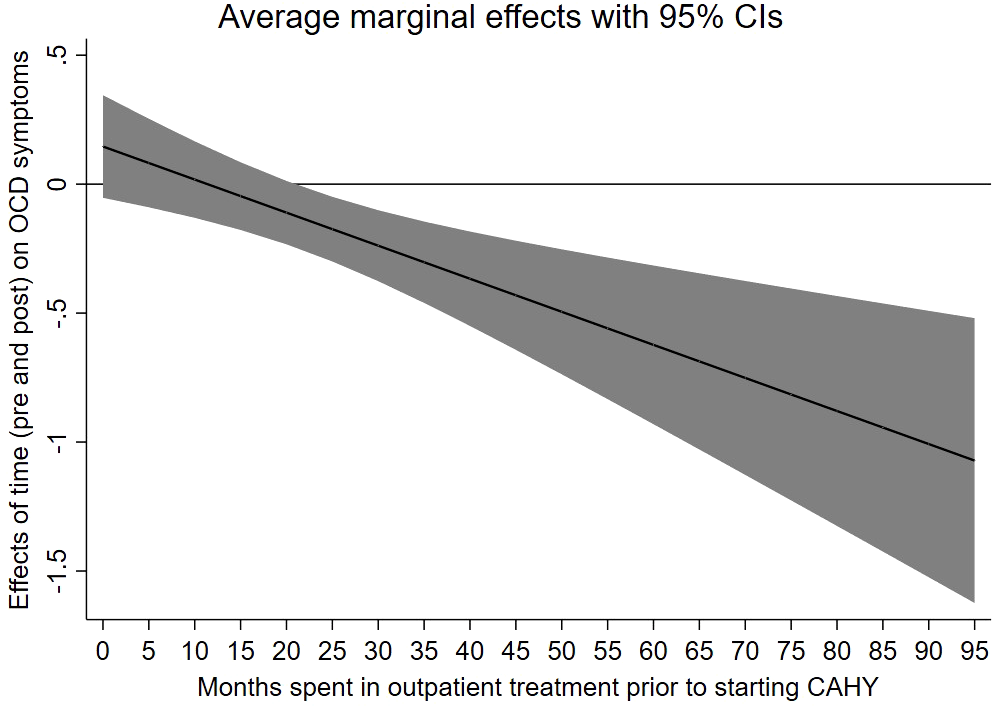
Association between prior treatment time in CAMHS and the subscale “OCD symptoms” on SCAS (parents ratings).

The analyses that included interaction terms revealed a statistically significant interaction between measurement point and time in outpatient care for obsessive compulsive disorder, *b* = -0.01, *p* <.01. This interaction is illustrated in **Figure 2** and shows that the reduction in OCD scores from pre- to post-measurement increases in relation to time spent in out-patient care prior to participation.

## Discussion

Treatment of anxiety disorders using physical activity is an under-used and under-researched area in child and adolescent mental health. The present study is one of very few studies that has examined the effect of a physical activity-based intervention targeting anxiety. To our knowledge, this is the first study to examine potential anxiety subgroup differential response, in a treatment seeking population of children and adolescents with mental health disorders. We found that while adolescents did not report any change on anxiety symptoms after participation in the treatment program, Confident, Active and Happy Youth (CAHY), their parents reported a significant reduction in anxiety symptoms in general and on the subscale assessing social phobia.

In terms of this positive change in parent reported anxiety symptoms, there are very few comparable studies. In the meta-analyses by Carter et al. (20), examining the effect of physical activity on anxiety, only a minority of studies included clinical populations (*n* = 4) and of these studies all included young adults (age < 25 years). However, allowing some difference in age for the sake of comparability, Carter et al. (20), found a moderate improvement in anxiety symptom reduction in the clinical studies, compared to a time and attention controlled group. The recent study by Phillippot et al. (24) included a clinical population of adolescents, in which they did not find a reduction of anxiety following treatment. Our results would seem to place themselves between the findings by Philipott et al. (24) and Carter et al. (20), indicating that a physical activity based intervention is viable and may contribute to positive symptom change. None of the studies included in the meta-analyses by Carter et al. (20), or the study by Philippot et al. (24) analyzed potential anxiety subgroups. Research including adult populations is also limited and more studies are needed in to order to investigate the role of physical activity in diverse anxiety disorders (13, 14). Preliminary findings indicate that physical activity could be effective in the treatment of panic disorder, generalized anxiety disorder, post-traumatic stress disorder (14) as well as social phobia (15).

Interestingly symptom change was found for the SCAS subscale social anxiety, but not for other SCAS subscales. CAHY is a group-based intervention, which has an explicit goal of exposing children and adolescents to some distress in terms of being physically active, cooperating with others and experiencing bodily sensations (feared stimulus) in front of others. This necessarily exposes the youth to some degree of social exposure training, and most likely more than an exposure to fears relating to an occupational compulsive disorder (OCD) or a separation anxiety. Thus, the intervention is likely more targeted to the underlying fears in social anxiety, rather than the core fears in OCD (i.e. fear of contamination) or a generalized anxiety (i.e. fear of worry). However, in terms of bodily sensations such as sweating, rapid heartbeats and breathlessness, these are common symptoms in panic attacks, which we would assume would also be experienced by the participants during CAHY sessions, thus some positive symptom change in this subgroup could be expected. However, no significant change was identified. In a systematic review, Frederiksen and colleagues (13) argue that physical exercise in combination with CBT so far has shown some positive results on symptom reduction in panic disorder. In our sample, only three participants were diagnosed with panic disorder (F41.0 in ICD-10), assuming only a few adolescents received treatment directed at this in CAMHS in addition to CAHY, which may explain lack of effect in this sub-group.

Our results indicated that youth and parent rated symptom changes from pre- to post-intervention were different. Several reasons may explain this difference. Both fear and avoidance behavior are core characteristics of anxiety disorders. While parents observe a reduction in avoidance behavior, youths may still exhibit great discomfort during social situations, which can explain the difference in parent and child reports. Also, questionnaires were filled out a short time after the last session in CAHY. This might also impact outcome on youth-reports, as it is suspected that youth are less aware of any change in their behavior at this time (rather than later). Thus, the lack of child-reported effect on anxiety symptoms may be explained by this lack of child awareness of own symptoms, and that awareness of change may take more time to occur (34). It can also be speculated whether exposure therapy of this type will increase symptoms for a period of time, before eventually decreasing as seen in other studies of anxiety treatment, examining long-term outcomes (35). Youth with social phobia often become “experts” in how they can avoid uncomfortable situations. During their time in CAHY, they are exposed to situations where they have to challenge themselves and their discomfort and interact with other youths and adults. These types of activities will increase their experience of social phobia, and many will exhibit physiological reactions. As a result, it can be expected that the youth reporting about their social phobia shortly after completing CAHY, will be less aware of any improvements.

Our analyses revealed no interaction between measurement point and age nor comorbidity. In regards to mental health comorbidity, our findings are in line with other studies examining the effect of comorbidity on treatment outcome, which found only minimal influence of comorbidity on anxiety outcome (36), although other studies have found Attention Deficit Hyperactive Disorder (ADHD) to have a more particular negative effect on anxiety change (37).

Our analyses indicated a significant effect of treatment time in CAMHS prior to starting CAHY on fear of physical injury (child scores) and OCD symptoms (parent scores). These findings were inverse, meaning the longer time in prior treatment in CAMHS was associated with an increase in fear of physical injury, whereas prior time in CAMHS was (assessed by parents) related to a decrease in OCD symptoms at post-treatment. These contradictory findings are difficult to explain and might reflect coincidences/bias due to the number of parameters estimated in the statistical models.

Exposure therapy is considered the first choice of treatment for anxiety disorders in youth (38, 39). However, as exposure treatment requires youth to engage in feared situations, some patients will be reluctant to attempt this treatment method. Physical activity is assumed to affect anxiety through exposure towards an avoided object and/or situation, as physical activity itself induces bodily sensations and reactions that otherwise might be interpreted as symptoms of anxiety and/or be negatively appraised. Exposure to such sensations and reactions in this setting is thus assumed to be associated with non-threatening experiences, and add to positive experiences with both the activity and peers, leading to normalization of such experiences and a more appropriate interpretation of these (40). Treatment based on physical activity might be less anxiety-provoking and thus lower the threshold for participation. CAHY as a supplement to therapy, might contribute to increasing the likelihood of compliance to other treatment methods.

With respect to future research, it would be interesting to examine the activity level of youths who participate in CAHY after treatment completion, and whether their overall activity level improves due to social cognition. Research finds that exercise enjoyment facilitates higher levels of physical activity among youths (41, 42). The activities in CAHY, including the last session, which is a group hike, are intended to be fun and enjoyable. Thus, it would be interesting to assess for improvements in social cognition among the participants.

## Strengths and limitations

The present study has several limitations and strengths. Our study included a clinical sample with a wide range of diverse diagnoses, offering a range of possible confounding variables. CAHY is a supplemental treatment program to treatment as usual in CAMHS, and we did not control for participant’s possible ongoing treatment and what type of treatment this might be during their participation in CAHY. This limits our ability to assess the effects of the intervention itself, versus any effects the youth had from ongoing treatment in CAMHS during and the intervention and the follow-up period. A future study should include a control group. However, inclusion of a clinical sample also adds knowledge of this type of intervention in a population group that is scarcely researched. Furthermore, the inclusion of participants using broad inclusion criteria and few exclusion criteria, adds external validity to the study. In keeping with our aim for this particular study (focusing on anxiety symptom change), we did not examine changes in other possible outcome variables, such as depressive or ADHD symptoms. These research questions are important to investigate in future work.

The study used a convenience sample, which limits the generalizability of the findings and the power of the findings. However, given the novelty of our study we assessed that our results are not only of interest towards a definitive trial, but other researchers working within the field. The fact the no studies, to our knowledge, within child and adolescent psychiatry has investigated potential sub-group treatment differences is a large gap in this research field, which is already limited.

Furthermore, the study was conducted during the COVID-19 pandemic. The pandemic itself and its associated restrictions may have affected interventions, as well as research outcomes during this period. Norway did enforce restrictions on society, beginning in the spring of 2020. However, restrictions were also fewer during the fall of 2020, data collection for this study started. Restrictions for children and adolescence were fewer, and less intrusive than for adults as the Norwegian Government abided by enforcing school, after-school activities and health care treatment as usual.

Our study incrementally adds support and research regarding the feasibility of incorporating physical activity in ordinary clinical practice and adding this tool to the therapist’s anxiety treatment toolbox, as an addition to “treatment as usual” in CAMHS. There are very few studies on this topic, and much more research is needed on the effects of physical activity among children and adolescents. Importantly, child developmental stages affect fear, and anxiety expressions, and studies on the effect of a physical activity program should also take into account these important differences between children. As indicated by our findings, physical activity is both feasible but also has differential effects on anxiety types.

## Conclusion

This study provides preliminary support to the use of interventions of physical activity in addition to treatment as usual in treating anxiety disorders in children and adolescents. Our findings support further work on this subject, which may be an important step towards meeting the increasing numbers of children and adolescents with mental health problems.

## Data availability statement

The raw data supporting the conclusions of this article will be made available by the authors, without undue reservation.

## Ethics statement

The studies involving humans were approved by Regionale komiteer for medisinsk og helsefaglig forskningsetikk (REK). The studies were conducted in accordance with the local legislation and institutional requirements. Written informed consent for participation in this study was provided by the participants’ legal guardians/next of kin.

## Author contributions

EA: Conceptualization, Methodology, Writing – original draft, Writing – review & editing. SS: Conceptualization, Methodology, Writing – original draft, Writing – review & editing. SH: Conceptualization, Data curation, Writing – review & editing, Formal analysis, Methodology, Software. AK: Conceptualization, Methodology, Project administration, Supervision, Validation, Writing – review & editing, Data curation, Investigation.

## References

[1] Krokstad S Weiss DA Krokstad MA Rangul V Kvaløy K Ingul JM. Divergent decennial trends in mental health according to age reveal poorer mental health for young people: repeated cross-sectional population-based surveys from the HUNT Study, Norway. BMJ Open. (2022) 12:e057654. doi: 10.1136/bmjopen-2021-057654 PMC9119156 35584877

[2] Solmi M Radua J Olivola M Croce E Soardo L Salazar de Pablo G. Age at onset of mental disorders worldwide: large-scale meta-analysis of 192 epidemiological studies. Mol Psychiatry. (2022) 27:281–95. doi: 10.1038/s41380-021-01161-7 PMC8960395 34079068

[3] Copeland WE Angold A Shanahan L Costello EJ. Longitudinal patterns of anxiety from childhood to adulthood: the Great Smoky Mountains Study. J Am Acad Child Adolesc Psychiatry. (2014) 53:21–33. doi: 10.1016/j.jaac.2013.09.017 24342383 PMC3939681

[4] Organization WH. Adolescent mental health in the European Region: Factsheet for World Mental Health Day 2018. Copenhagen, Denmark: World Health Organization. Regional Office for Europe (2018).

[5] Racine N McArthur BA Cooke JE Eirich R Zhu J Madigan S. Global prevalence of depressive and anxiety symptoms in children and adolescents during COVID-19: A meta-analysis. JAMA Pediatr. (2021) 175:1142–50. doi: 10.1001/jamapediatrics.2021.2482 PMC8353576 34369987

[6] Deborah Omoleye D Olubukola Abidakun O Oluwadamilola Akinje R Hannah Ademuyiwa O Mofoluwaso Fasogbon B. A review of the effects of the COVID-19 pandemic on children and ado-lescents’ Mental health. Curr Pediatr Rev. (2023) 19:1–8. 10.2174/1573396319666230213104546 36788690

[7] Necho M Tsehay M Birkie M Biset G Tadesse E. Prevalence of anxiety, depression, and psychological distress among the general population during the COVID-19 pandemic: a systematic review and meta-analysis. Int J Soc Psychiatry. (2021) 67:892–906. doi: 10.1177/00207640211003121 33794717

[8] Longest K Kang J-A. Social media, social support, and mental health of young adults during COVID-19. Front Communication. (2022) 7. doi: 10.3389/fcomm.2022.828135

[9] Benton TD Boyd RC Njoroge WFM. Addressing the global crisis of child and adolescent mental health. JAMA Pediatr. (2021) 175:1108–10. doi: 10.1001/jamapediatrics.2021.2479 34369961

[10] Wergeland GJH Riise EN Ost LG. Cognitive behavior therapy for internalizing disorders in children and adolescents in routine clinical care: A systematic review and meta-analysis. Clin Psychol Rev. (2021) 83:101918. doi: 10.1016/j.cpr.2020.101918 33186776

[11] Raballo A Poletti M Valmaggia L McGorry PD. Editorial Perspective: Rethinking child and adolescent mental health care after COVID-19. J Child Psychol Psychiatry. (2021) 62:1067–9. doi: 10.1111/jcpp.13371 33368236

[12] Weisz JR Kuppens S Ng MY Eckshtain D Ugueto AM Vaughn-Coaxum R. What five decades of research tells us about the effects of youth psychological therapy: A multilevel meta-analysis and implications for science and practice. Am Psychol. (2017) 72:79. doi: 10.1037/a0040360 28221063

[13] Frederiksen KP Stavestrand SH Venemyr SK Sirevag K Hovland A. Physical exercise as an add-on treatment to cognitive behavioural therapy for anxiety: a systematic review. Behav Cognit Psychoth. (2021) 49:626–40. doi: 10.1017/S1352465821000126 33678210

[14] Strohle A. Physical activity, exercise, depression and anxiety disorders. J Neural Transm (Vienna). (2009) 116:777–84. doi: 10.1007/s00702-008-0092-x 18726137

[15] Merom D Phongsavan P Wagner R Chey T Marnane C Steel Z. Promoting walking as an adjunct intervention to group cognitive behavioral therapy for anxiety disorders–a pilot group randomized trial. J Anxiety Disord. (2008) 22:959–68. doi: 10.1016/j.janxdis.2007.09.010 17988832

[16] Broeren S Muris P. The relation between cognitive development and anxiety phenomena in children. J Child Fam Stud. (2009) 18:702–9. doi: 10.1007/s10826-009-9276-8 PMC2765654 19855850

[17] Ollendick TH King NJ Muris P. Fears and phobias in children: phenomenology, epidemiology, and aetiology. ACAMH. (2002) 7:98–106. doi: 10.1111/1475-3588.00019

[18] Puccinelli PJ da Costa TS Seffrin A de Lira CAB Vancini RL Nikolaidis PT. Reduced level of physical activity during COVID-19 pandemic is associated with depression and anxiety levels: an internet-based survey. BMC Public Health. (2021) 21:425. doi: 10.1186/s12889-021-10684-1 33648487 PMC7919983

[19] Singh B Olds T Curtis R Dumuid D Virgara R Watson A. Effectiveness of physical activity interventions for improving depression, anxiety and distress: an overview of systematic reviews. Br J Sports Med. (2023) 57(18):bjsports-2022-106195. doi: 10.1136/bjsports-2022-106195 PMC10579187 36796860

[20] Carter T Pascoe M Bastounis A Morres ID Callaghan P Parker AG. The effect of physical activity on anxiety in children and young people: a systematic review and meta-analysis. J Affect Disord. (2021) 285:10–21. doi: 10.1016/j.jad.2021.02.026 33618056

[21] Recchia F Bernal JDK Fong DY Wong SHS Chung PK Chan DKC. Physical activity interventions to alleviate depressive symptoms in children and adolescents: A systematic review and meta-analysis. JAMA Pediatr. (2023) 177:132–40. doi: 10.1001/jamapediatrics.2022.5090 PMC9857695 36595284

[22] Axelsdottir B Biedilae S Sagatun A Nordheim LV Larun L. Review: Exercise for depression in children and adolescents - a systematic review and meta-analysis. J Child Adolesc Ment Health. (2021) 26:347–56. doi: 10.1111/camh.12438 33277972

[23] Bailey AP Hetrick SE Rosenbaum S Purcell R Parker AG. Treating depression with physical activity in adolescents and young adults: a systematic review and meta-analysis of randomised controlled trials. Psychol Med. (2018) 48:1068–83. doi: 10.1017/S0033291717002653 28994355

[24] Philippot A Dubois V Lambrechts K Grogna D Robert A Jonckheer U. Impact of physical exercise on depression and anxiety in adolescent inpatients: A randomized controlled trial. J Affect Disord. (2022) 301:145–53. doi: 10.1016/j.jad.2022.01.011 35007642

[25] Firth J Siddiqi N Koyanagi A Siskind D Rosenbaum S Galletly C. The Lancet Psychiatry Commission: a blueprint for protecting physical health in people with mental illness. Lancet Psychiatry. (2019) 6:675–712. doi: 10.1016/S2215-0366(19)30132-4 31324560

[26] Kodal A Muirhead F Reilly JJ Wergeland GJ Thorsen PJB Bovim LPV. Feasibility of a physical activity intervention for children and adolescents with anxiety and depression. Pilot Feasibility Stud. (2024) 10(49):1–12. doi: 1186/s40814-024-01466-8 38443992 10.1186/s40814-024-01466-8 PMC10913538

[27] Skivington K Matthews L Simpson SA Craig P Baird J Blazeby JM. A new framework for developing and evaluating complex interventions: update of Medical Research Council guidance. BMJ. (2021) 374:n2061. doi: 10.1136/bmj.n2061 34593508 PMC8482308

[28] Kodal A Muirhead F Reilly JJ Wergeland GJH Thorsen PJB Bovim LP. Development and feasibility testing of a physical activity intervention for youth with anxiety and depression: a study protocol. Pilot Feasibility Stud. (2022) 8:48. doi: 10.1186/s40814-022-01010-6 35236419 PMC8889653

[29] Spence SH. A measure of anxiety symptoms among children. Behav Res Ther. (1998) 36:545–66. doi: 10.1016/S0005-7967(98)00034-5 9648330

[30] Kenward MG Roger JH. Small sample inference for fixed effects from restricted maximum likelihood. Biometrics. (1997) 53:983–97. doi: 10.2307/2533558 9333350

[31] Schafer JL Graham JW. Missing data: Our view of the state of the art. psychol Methods. (2002) 7:147–77. doi: 10.1037//1082-989X.7.2.147 12090408

[32] Molenberghs G Thijs H Jansen I Beunckens C Kenward MG Mallinckrodt C. Analyzing incomplete longitudinal clinical trial data. Biostatistics. (2004) 5:445–64. doi: 10.1093/biostatistics/kxh001 15208205

[33] StataCorp. Stata statistical software: Release 17. College Station, TX: StataCorp LLC (2021).

[34] Schniering CA Hudson JL Rapee RM. Issues in the diagnosis and assessment of anxiety disorders in children and adolescents. Clin Psychol Rev. (2000) 20:453–78. doi: 10.1016/S0272-7358(99)00037-9 10832549

[35] Kodal A Fjermestad K Bjelland I Gjestad R Ost LG Bjaastad JF. Long-term effectiveness of cognitive behavioral therapy for youth with anxiety disorders. J Anxiety Disord. (2018) 53:58–67. doi: 10.1016/j.janxdis.2017.11.003 29195188

[36] Ollendick TH Jarrett MA Grills-Taquechel AE Hovey LD Wolff JC. Comorbidity as a predictor and moderator of treatment outcome in youth with anxiety, affective, attention deficit/hyperactivity disorder, and oppositional/conduct disorders. Clin Psychol Review. (2008) 28:1447–71. doi: 10.1016/j.cpr.2008.09.003 18973971

[37] Halldorsdottir T Ollendick TH. Comorbid ADHD: implications for the treatment of anxiety disorders in children and adolescents. Cognit Behav Pract. (2014) 21:310–22. doi: 10.1016/j.cbpra.2013.08.003

[38] Craske MG Treanor M Conway CC Zbozinek T Vervliet B. Maximizing exposure therapy: an inhibitory learning approach. Behav Res Ther. (2014) 58:10–23. doi: 10.1016/j.brat.2014.04.006 24864005 PMC4114726

[39] Kaczkurkin AN Foa EB. Cognitive-behavioral therapy for anxiety disorders: an update on the empirical evidence. Dialogues Clin Neurosci. (2015) 17:337–46. doi: 10.31887/DCNS.2015.17.3/akaczkurkin PMC4610618 26487814

[40] Kandola A Vancampfort D Herring M Rebar A Hallgren M Firth J. Moving to beat anxiety: epidemiology and therapeutic issues with physical activity for anxiety. Curr Psychiatry Rep. (2018) 20:63. doi: 10.1007/s11920-018-0923-x 30043270 PMC6061211

[41] Simpson P Howie E Williams S Neil A Morris S Ng L. The relationship between competence and enjoyment with physical activity in children: it depends on the dependent variable. JSAMS. (2017) 20:e77. doi: 10.1016/j.jsams.2017.01.027

[42] Chen H Sun H Dai J. Peer support and adolescents’ physical activity: The mediating roles of self-efficacy and enjoyment. J Pediatr Psychol. (2017) 42:569–77. doi: 10.1093/jpepsy/jsw103 28158660

